# Detection and characterization of a novel marine birnavirus isolated from Asian seabass in Singapore

**DOI:** 10.1186/s12985-019-1174-0

**Published:** 2019-05-28

**Authors:** Jing Chen, Xinyu Toh, Jasmine Ong, Yahui Wang, Xuan-Hui Teo, Bernett Lee, Pui-San Wong, Denyse Khor, Shin-Min Chong, Diana Chee, Alvin Wee, Yifan Wang, Mee-Keun Ng, Boon-Huan Tan, Taoqi Huangfu

**Affiliations:** 1 Centre for Animal & Veterinary Sciences, Professional and Scientific Services, Animal and Veterinary Service, National Parks Board (NParks), 1 Cluny Road, Singapore, 259569 Singapore; 2 Singapore Immunology Network, Agency for Science, Technology and Research (A*STAR), 8A Biomedical Grove, Singapore, 138648 Singapore; 3 DSO National Laboratories, 27 Medical Drive, Singapore, 117510 Singapore

**Keywords:** *Lates calcarifer* Birnavirus (LCBV), Asian seabass, Next-generation sequencing (NGS)

## Abstract

**Background:**

*Lates calcarifer*, known as seabass in Asia and barramundi in Australia, is a widely farmed species internationally and in Southeast Asia and any disease outbreak will have a great economic impact on the aquaculture industry. Through disease investigation of Asian seabass from a coastal fish farm in 2015 in Singapore, a novel birnavirus named *Lates calcarifer* Birnavirus (LCBV) was detected and we sought to isolate and characterize the virus through molecular and biochemical methods.

**Methods:**

In order to propagate the novel birnavirus LCBV, the virus was inoculated into the Bluegill Fry (BF-2) cell line and similar clinical signs of disease were reproduced in an experimental fish challenge study using the virus isolate. Virus morphology was visualized using transmission electron microscopy (TEM). Biochemical analysis using chloroform and 5-Bromo-2′-deoxyuridine (BUDR) sensitivity assays were employed to characterize the virus. Next-Generation Sequencing (NGS) was also used to obtain the virus genome for genetic and phylogenetic analyses.

**Results:**

The LCBV-infected BF-2 cell line showed cytopathic effects such as rounding and granulation of cells, localized cell death and detachment of cells observed at 3 to 5 days’ post-infection. The propagated virus, when injected intra-peritoneally into naïve Asian seabass under experimental conditions, induced lesions similar to fish naturally infected with LCBV. Morphology of LCBV, visualized under TEM, revealed icosahedral particles around 50 nm in diameter. Chloroform and BUDR sensitivity assays confirmed the virus to be a non-enveloped RNA virus. Further genome analysis using NGS identified the virus to be a birnavirus with two genome segments. Phylogenetic analyses revealed that LCBV is more closely related to the *Blosnavirus* genus than to the *Aquabirnavirus* genus within the *Birnaviridae* family.

**Conclusions:**

These findings revealed the presence of a novel birnavirus that could be linked to the disease observed in the Asian seabass from the coastal fish farms in Singapore. This calls for more studies on disease transmission and enhanced surveillance programs to be carried out to understand pathogenicity and epidemiology of this novel virus. The gene sequences data obtained from the study can also pave way to the development of PCR-based diagnostic test methods that will enable quick and specific identification of the virus in future disease investigations.

## Background

*Lates calcarifer,* known as seabass in Asia and barramundi in Australia, is a member of the family *Centropomidae* that is widely farmed in Southeast Asia, including Singapore [1]. Asian seabass are ideal candidates for aquaculture due to their wide physiological tolerances and rapid growth. These Asian seabasses can grow to a harvestable size (350 g – 3 kg) in 6 months to 2 years. Even though Asian seabass is a robust species, they are susceptible to a wide range of diseases under rearing conditions and disease outbreaks can have important effects on commercial production. There are at least two new viruses such as Scale Drop Disease Virus (SDDV) and *Lates calcarifer* Herpes Virus (LCHV) which have been detected and isolated from diseased Asian seabass in recent years [2, 3].

In early 2015, periodic episodes of mortality occurred in a coastal Asian seabass farm in Singapore. Diseased seabass from this farm were submitted to the Animal Health Laboratory, Agri-Food & Veterinary Authority of Singapore (AVA) as part of disease investigation. Gross examination of the diseased fish revealed multifocal “white patch” lesions on the body. Hence, a viral agent was suspected to be involved in the disease outbreak.

Routine diagnostics for known common viral pathogens (megalocytiviruses and viral nervous necrosis virus) did not yield any positive detection. Further attempts to identify emerging viral pathogens of seabass such as SDDV, were unsuccessful. Further investigation including cell culture, transmission electron microscopy (TEM) observation, chloroform and 5-Bromo-2′-deoxyuridine (BUDR) treatments and Next-Generation Sequencing (NGS) identified a novel birnavirus. The novel birnavirus is named as *Lates calcarifer* Birnavirus (LCBV) which can be classified within the *Birnaviridae* virus family. Since its first detection in early 2015, the birnavirus has been detected and isolated from diseased Asian seabass till now.

These findings provide critical information about the presence of a novel birnavirus that could be linked to disease observed in the Asian seabass from the coastal fish farm. This re-iterates the importance for disease transmission studies and enhanced surveillance programs to minimize economic losses in aquaculture. New diagnostic methods for LCBV can also be developed based on the gene sequences to facilitate detection and quick disease diagnosis.

## Methods

### Post-mortem examination

Three diseased Asian seabass from a coastal farm were submitted to the Animal Health Laboratory, AVA for examination on 10 February 2015. The submission was identified as case A17/2/15. The fishes had an average bodyweight of 272 g and average body length of 23.5 cm. Samples of skin, muscle, eye, brain, gills, heart, liver, pancreas, kidney and spleen were taken for histopathology, bacteriology and virology examination. On a separate occasion, diseased Asian seabass were submitted for examination under case A11/6/17 on 06 June 2017. The five Asian seabass submitted had an average bodyweight of 23.1 g and an average body length of 10.4 cm. Similar laboratory examinations were carried out.

### Cell culture isolation

Virus isolation in Bluegill fry (BF-2) cell line was carried out using the organ pools of kidney, liver and spleen collected from the diseased fishes under cases A17/2/15 and A11/6/17. The organ homogenates were first filtered through a membrane filter with a pore size of 0.45 μm (Millipore) before inoculating onto the pre-cultured BF-2 cell monolayer. The cells were cultured at 25+/− 2 °C for 1–2 days in 25cm^2^ tissue culture flasks in 5 ml of growth medium (Minimal Essential Media (MEM) with L-glutamine, supplemented with 10% fetal bovine serum, 200 IU/mL penicillin G, 200 μg/mL streptomycin sulphate, 0.035% bicarbonate). Growth medium was removed from the flasks before inoculation of the organ homogenates. After an incubation of 1 h, culture medium (MEM with L-glutamine, supplemented with 2% fetal bovine serum, 200 IU/ml penicillin G, 200 μg/ml streptomycin sulphate, 0.07% bicarbonate, and 0.5% of 1 M 4-(2-hydroxyethyl)-1-piperazineethanesulfonic acid (HEPES buffer) was added to the flasks. The flasks were then incubated at 25+/− 2 °C, with daily observation for cytopathic effect (CPE). Virus infectivity was assayed using 96-well microplates containing monolayers of BF-2 cells. The titre was determined by 50% tissue culture infective dose (TCID_50_) assay with endpoints calculated by the method of Reed and Mu¨ench [4].

### Purification of the virus

The infected BF-2 cell culture flasks were frozen and thawed 3 times before centrifugation of the cell culture fluid at 3500 g for 10mins at 4 °C. The supernatant was collected and filtered through a 0.22 μm syringe filter. The filtered supernatant was loaded on top of a 30% sucrose cushion in a centrifuge tube before centrifugation at 36,000 rpm (~ 100,000 g) using Beckman Type 45 rotor for 2 h at 4 °C to pellet the virus. The virus pellet was dissolved in 0.5–1 mL TNE buffer (10 mM Tris-HCL, 100 mM NaCL, 10 mM EDTA, pH 7.5) and aliquots of purified virus were stored at − 70 °C.

### Examination by transmission Electron microscopy

BF-2 cells were infected with LCBV. Two days after infection, infected cells were pelleted in 1X phosphate buffered solution (PBS) (Invitrogen, USA) and fixed with 4% paraformaldehyde. The infected cell pellets were next subjected to dehydration through a series of increasing concentrations of ethanol at room temperature. The cell pellets were embedded with the resin mixture (26% v/v Epon 812, 58% v/v Dodecenyl succinic anhydride) (Electron Microscopy Sciences, USA), and 16% v/v Araldite 502 (Electron Microscopy Sciences, USA). The polymerized samples were sectioned with an ultra-microtome (Leica, USA), the sections were double-stained with lead citrate (Electron Microscopy Sciences, USA) and uranyl acetate (SPI Supplies, USA), and viewed under the transmission electron microscope (TEM) (Model 1010, JEOL). The electron micrographs were captured with the Gatan Charge-Coupled Device (CCD) Camera attached to the TEM.

### Sensitivity to chloroform and BUDR

The 6th passage of the cell culture fluid from case A17/2/15 was mixed thoroughly with 10% (v/v) chloroform and incubated for 10mins at room temperature to let the chloroform react with the virus [5–7]. The mixture is centrifuged for 10mins at 1500–2000 rpm, 2–8 °C and the uppermost layer was used for measurement of the virus infectivity on day 7. A known enveloped virus was included in the experiment as control.

Similarly, monolayer cultures of BF-2 cells, infected with the 6th passage of the cell culture fluid, were exposed to BUDR or to control medium [5, 7] and incubated at 25 °C. The virus infectivity was measured at day 7. A known DNA virus was included in the experiment as control.

### Next-generation sequencing (NGS)

The total RNA was extracted from the purified virus of case A17/2/15 using Direct-zol™ RNA MiniPrep Plus kit and cell culture fluid of the 5th passage of case A11/6/17 using QIAamp Viral RNA Extraction Kit. The extracted RNA was used as an input for NGS on an Illumina MiSeq platform. RNA library preparation steps were performed at AIT Biotech Pte Ltd. using the TruSeq Total RNA Sample Preparation kit without ribosomal RNA depletion, according to manufacturer’s instructions. Reads that were generated from the MiSeq Nano flowcell were assembled into contigs using the Trinity program. Assembled contigs were used in BLAT to search against the NCBI RefSeq and GenBank viral sequences to align the contig sequences with the available viral sequences present in the library.

### Phylogenetic analysis

A total of 1134 amino acid (aa) positions was used for the phylogenetic analysis. The amino acid sequence of contig DN13010 (1090 aa) generated from NGS data analysis of case A11/6/17 was translated from the nucleotide sequence using ExPASy Proteomic server. The deduced amino acid sequence was aligned and compared with major capsid protein (VP2) from various viruses using BioEdit v7.1.3 [8]. These sequences were aligned using ClustalX2 and a phylogenetic tree was constructed through maximum likelihood analysis. Using ModelGenerator v0.85 [9], the best-fitting evolutionary model under the Akaike Information Criterion was the Le and Gascuel model [10]. The maximum likelihood analysis was ran using RaxML v8.2.5 with 500 bootstraps [11]. The tree was determined to have converged after 450 replicates by the bootstrap convergence criterion.

### Experimental fish infection

An experimental fish challenge study was carried out to test the Koch’s postulates: to determine whether the fish injected with LCBV would display similar clinical signs and disease, to re-isolate, grow and characterize the causative agent [12, 13].

Asian seabass fingerlings (around 3 months old, 20-30 g in weight) were obtained from Marine Aquaculture Centre, AVA. The fish were maintained under experimental conditions at 28 °C and fed with commercially prepared dry pellets. Twelve fish were sacrificed to test if the fish are free from known fish pathogens. Twenty-four fish were injected using the intra-peritoneal (i.p.) route with 0.1 mL of infected BF-2 cell culture supernatant (case A17/2/15, 6p) containing two different virus concentrations (3 × 10^5^ and 3 × 10^6^ TCID_50_/mL). Twelve fishes were injected with 0.1 mL of uninfected BF-2 cell culture supernatant as control group. Supernatant of the cell culture was prepared by centrifugation at 2000 g for 15mins and filtered with 0.45 μm syringe filter before inoculation. The fish and water quality were monitored daily. Dead and moribund fish were collected when they have been found. All the remaining fish were euthanised at end of week 5 after injection. The liver, spleen and kidney were kept for Polymerase Chain Reaction (PCR), histopathology, cell culture isolation and routine bacteriology examination.

## Results

### Isolation of LCBV from infected cell culture

**Fig. 1:**
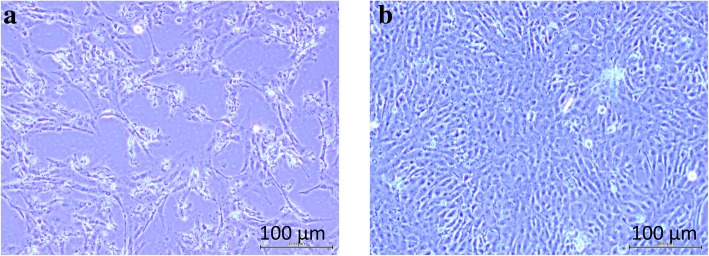
BF-2 cells infected with case A17/2/15 organ suspension showed CPE. **a** Day 2 post-infection of cells inoculated with case A17/2/15 organ suspension demonstrated CPE such as rounding and detachment from surface; **b** Uninfected BF-2 cell monolayer showed no evident CPE

Internal organs such as kidney, liver and spleen from cases A17/2/15 and A11/6/17 were collected for virus isolation using BF-2 cell culture. The BF-2 cells showed CPE within 3 to 5 days’ post-inoculation of samples of cases A17/2/15 and A11/6/17. Characteristic CPE observations include rounding and granulation of cells, localized cell death and detachment of cells (Fig. 1a). The control BF-2 cell monolayer remained normal and intact throughout the incubation (Fig. 1b). Subsequent passages (up to 6 passages) of the cell culture fluid to a fresh BF-2 cell monolayer showed a similar CPE, suggesting that the described CPE was due to the presence of an infectious viral agent. The titre of 6th passage cell culture fluid of case A17/2/15 was determined to be 10^8.8^ TCID_50_/mL.

### Morphological identification of LCBV

**Fig. 2:**
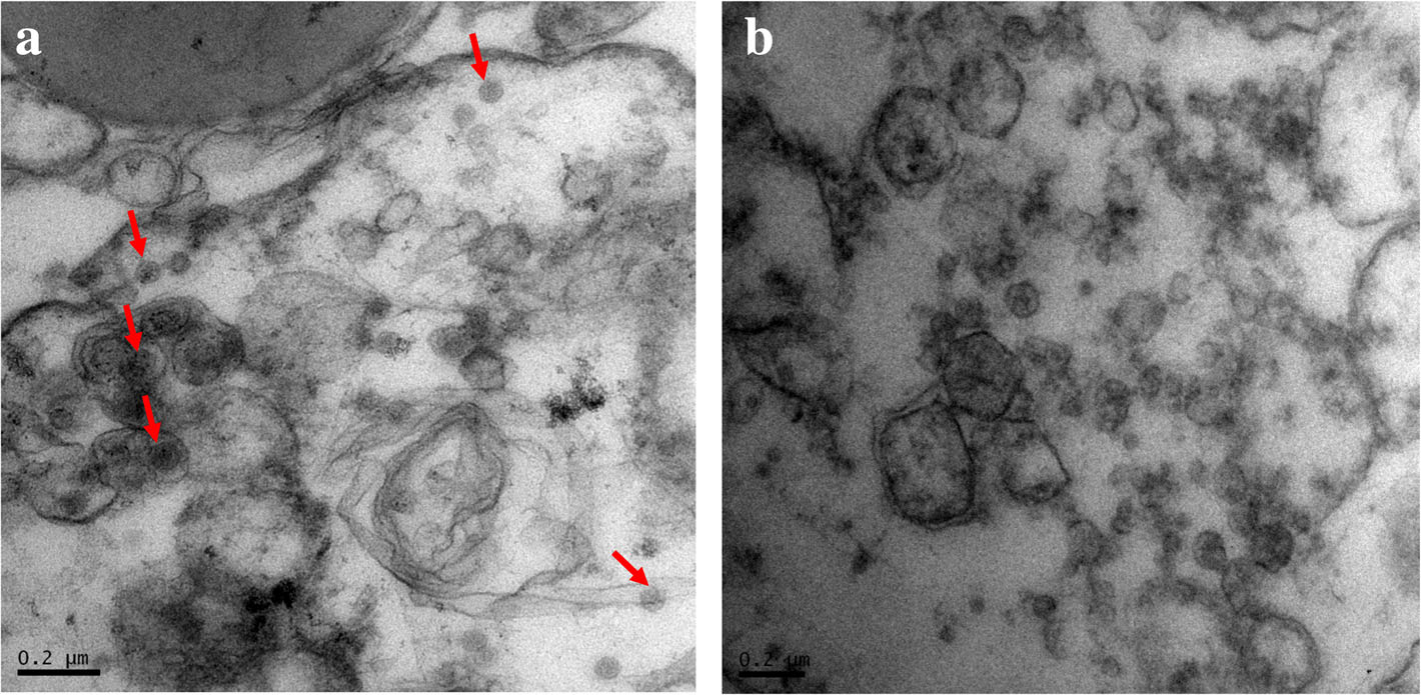
Transmission electron microscope observation of BF-2 cells infected with case A17/2/15 organ suspension. **a** Virus particles found in cells infected with case A17/2/15 organ suspension were found to be ~ 50 nm in diameter with icosahedral shape; **b** No virus particle can be seen in uninfected BF-2 cell control

TEM examination provided evidence for the presence of a viral infection in BF-2 cell line after inoculation with case A17/2/15 organ suspension. The TEM analysis revealed the presence of electron-dense particles of around 50 nm in diameter with an icosahedral shape in the infected BF-2 cell pellet (Fig. 2a). The virions were abundant in the infected BF-2 cell pellet whereas virions could not be seen in the control uninfected BF-2 cell pellet (Fig. 2b).

### Sensitivity of LCBV to chloroform and BUDR

To characterize the infectious viral agent, the supernatant of BF-2 cells infected by case A17/2/15 (6th passage) were exposed to chloroform and the effect of chloroform treatment on infectivity were measured by infectivity assays. A loss of infectivity of less than 1 log_10_ was observed after chloroform treatment, suggesting that the infectious agent from diseased seabass was a non-enveloped virus (Table 1). On the other hand, a control virus (Frog Virus 3) with an enveloped structure showed total reduction in infectivity.

**Table 1:** Chloroform and BUDR sensitivity test

Treatment	Samples	TCID_50_/ml without treatment	TCID_50_/ml with treatment	Infectivity reduction	Conclusion
Chloroform	Case A17/2/15 6p	10^5.33^	10^4.83^	10^0.5^ < 1 log10	Non-enveloped Virus
	Frog Virus 3 (Control)	10^5^	NA	No infectivity	Enveloped Virus
BUDR	Case A17/2/15 6p	10^5.5^	10^5.33^	10^0.17^ < 1 log10	RNA Virus
	Frog Virus 3 (Control)	10^5.5^	NA	No infectivity	DNA Virus

To further determine the genome properties of the infectious agent, BUDR treatment was also performed using the supernatant of BF-2-infected cells of case A17/2/15 (6th passage) and the effects of BUDR treatment on DNA replication were measured. A loss of infectivity of less than 1 log_10_ was observed after BUDR treatment, indicating that the virus had a RNA genome (Table 1). In contrast, the control virus with a DNA genome showed total reduction in infectivity.

### Molecular characterization of LCBV

NGS was performed for whole viral genome characterization of sample from case A17/2/15. Sequencing reads were de novo assembled into contigs using Trinity. The assembled contigs were then used by BLAT to search against the NCBI RefSeq and GenBank collection of viral sequences to align the contigs with the available viral sequences in the library. Most of the contigs did not match to any genes of aquatic origin with the exception of 2 contigs which were of interest in our study: DN177 (2502 bp) and DN154 (2744 bp) that mapped onto the partial sequences of *Channa lucius* virus genomic segment A and *Channa lucius* virus genomic segment B, respectively. The DN154 contig showed ~ 69% identity over 1638 bp with *Channa lucius* VP1 gene for RNA-dependent RNA polymerase.

*Channa lucius* virus, also known as Blotched snakehead virus (BSNV), belongs to the *Blosnavirus* genus within the *Birnaviridae* family which comprises of four genera: *Aquabirnavirus, Avibirnavirus, Blosnavirus and Entomobirnavirus* [14, 15]. Birnaviruses are characterized by a bi-segmented double-stranded RNA genome enclosed within a non-enveloped, icosahedral capsid of about 50-60 nm in diameter. The larger genome segment (Segment A) of about 3000 base pairs (bp) encodes for a precursor polyprotein that is subsequently cleaved into 3 viral proteins (pVP2, NS and VP3). Of which, the pVP2 protein can be further processed into the major capsid protein VP2. The smaller genome segment (Segment B) of about 2500 base pairs (bp) encodes for the RNA-dependent RNA polymerase. Therefore, contig assembly resulted in identification of the genome sequence of a putative birnavirus that has a similar genome organisation to BSNV.

NGS was also performed on sample from case A11/6/17. A total of 250,175 reads was used to construct contig DN13010 (3527 bp), and 358,495 reads was used to construct contig DN14359 (2731 bp). There were about only 18–21 nucleotide differences between the contigs of DN177 with DN13010, and DN154 with DN14359, suggesting the presence of the same birnavirus in both cases of A17/2/15 and A11/6/17. The nucleotide sequences of contigs DN13010 and DN14359 were uploaded to GenBank with accession numbers MK103419 and MK103420 respectively.

### Phylogenetic analysis of LCBV

**Fig. 3:**
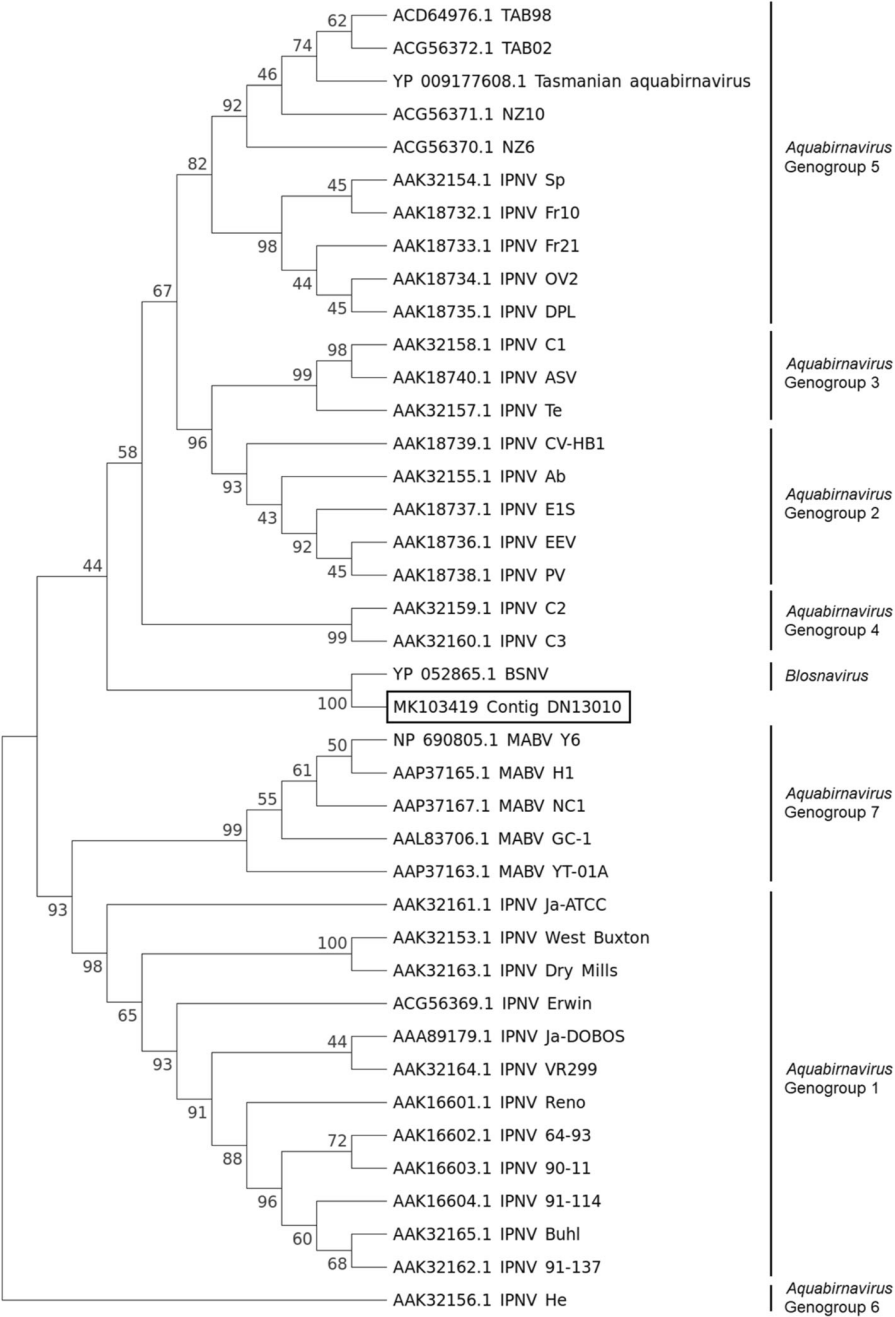
The evolutionary history of the VP2 protein of viruses in the *Aquabirnavirus* and *Blosnavirus* genera was inferred by using the maximum likelihood method based on the Le and Gascuel model. A total of 1134 amino acid positions was used for the phylogenetic analysis. The bootstrap consensus tree inferred from 500 replicates is taken to represent the evolutionary history of the taxa analyzed. Numbers presented at each branch point represent bootstrap percentage from 500 replicates. Accession numbers of each amino acid sequence used are indicated at the beginning of each sequence. (IPNV – Infectious pancreatic necrosis virus; MABV – marine aquabirnavirus)

Phylogenetic analysis was also exploited to characterize LCBV based on NGS results of case A11/6/17. Phylogenetic analysis of the VP2 revealed that LCBV was grouped together with BSNV, and is in a separate group from infectious pancreatic necrosis virus (IPNV) and the other viruses in the *Aquabirnavirus* genus (Fig. 3). BSNV is classified in the *Birnaviridae* family based on its biochemical characteristics, cross-neutralisation assays [16]. The clustering of contig DN13010 together with the VP2 of BSNV suggests that LCBV is more related to the *Blosnavirus* genus than to the *Aquabirnavirus* genus*.* Furthermore, contig DN13010 shares the highest percentage identity of 48.4% with the VP2 of BSNV as compared to the other birnaviruses (Table 2). Nonetheless, further characterisation work is required for confirmation of their relatedness.

**Table 2:** Amino acid percentage identity between contig DN13010 and VP2 of various viruses in the *Aquabirnavirus* and *Blosnavirus* genera

Sequence	Genus	Genogroup	Percentage identity (%)
MK103419 Contig DN13010	To be determined	To be determined	ID
YP_52865.1 BSNV	*Blosnavirus*	*Blosnavirus*	48.4
ACG56369.1 Erwin	*Aquabirnavirus*	1	40.5
AAK16601.1 Reno	*Aquabirnavirus*	1	39.6
AAK16602.1 64–93	*Aquabirnavirus*	1	39.4
AAK16603.1 90–11	*Aquabirnavirus*	1	39.4
AAK16604.1 91–114	*Aquabirnavirus*	1	39.4
AAK32153.1 West Buxton	*Aquabirnavirus*	1	35.7
AAK32163.1 Dry Mills	*Aquabirnavirus*	1	35.7
AAK32161.1 Ja-ATCC	*Aquabirnavirus*	1	35.7
AAK32164.1 VR299	*Aquabirnavirus*	1	35.5
AAK32162.1 91–137	*Aquabirnavirus*	1	35.5
AAA89179.1 Ja-DOBOS	*Aquabirnavirus*	1	35.3
AAK32165.1 Buhl	*Aquabirnavirus*	1	35.3
AAK18737.1 E1S	*Aquabirnavirus*	2	40.0
AAK18738.1 PV	*Aquabirnavirus*	2	39.8
AAK18736.1 EEV	*Aquabirnavirus*	2	39.6
AAK18739.1 CV-HB1	*Aquabirnavirus*	2	39.4
AAK32155.1 Ab	*Aquabirnavirus*	2	34.7
AAK18740.1 ASV	*Aquabirnavirus*	3	41.1
AAK32158.1 C1	*Aquabirnavirus*	3	35.7
AAK32157.1 Te	*Aquabirnavirus*	3	35.1
AAK32159.1 C2	*Aquabirnavirus*	4	35.6
AAK32160.1 C3	*Aquabirnavirus*	4	35.0
AAK18732.1 Fr10	*Aquabirnavirus*	5	41.3
AAK18735.1 DPL	*Aquabirnavirus*	5	41.3
AAK18733.1 Fr21	*Aquabirnavirus*	5	41.1
AAK18734.1 OV2	*Aquabirnavirus*	5	41.1
ACG56370.1 NZ6	*Aquabirnavirus*	5	41.1
ACG56371.1 NZ10	*Aquabirnavirus*	5	41.1
ACG56372.1 TAB02	*Aquabirnavirus*	5	41.0
YP_9177608.1 Tasmanian aquabirnavirus	*Aquabirnavirus*	5	35.8
AAK32154.1 Sp	*Aquabirnavirus*	5	35.8
ACD64976.1 TAB98	*Aquabirnavirus*	5	35.7
AAK32156.1 He	*Aquabirnavirus*	6	35.0
NP_690805.1 MABV Y6	*Aquabirnavirus*	7	35.9
AAP37165.1 H1	*Aquabirnavirus*	7	35.8
AAP37167.1 NC1	*Aquabirnavirus*	7	35.8
AAP37163.1 YT-01A	*Aquabirnavirus*	7	35.8
AAL83706.1 GC-1	*Aquabirnavirus*	7	35.4

### Experimental fish infection with LCBV

**Fig. 4:**
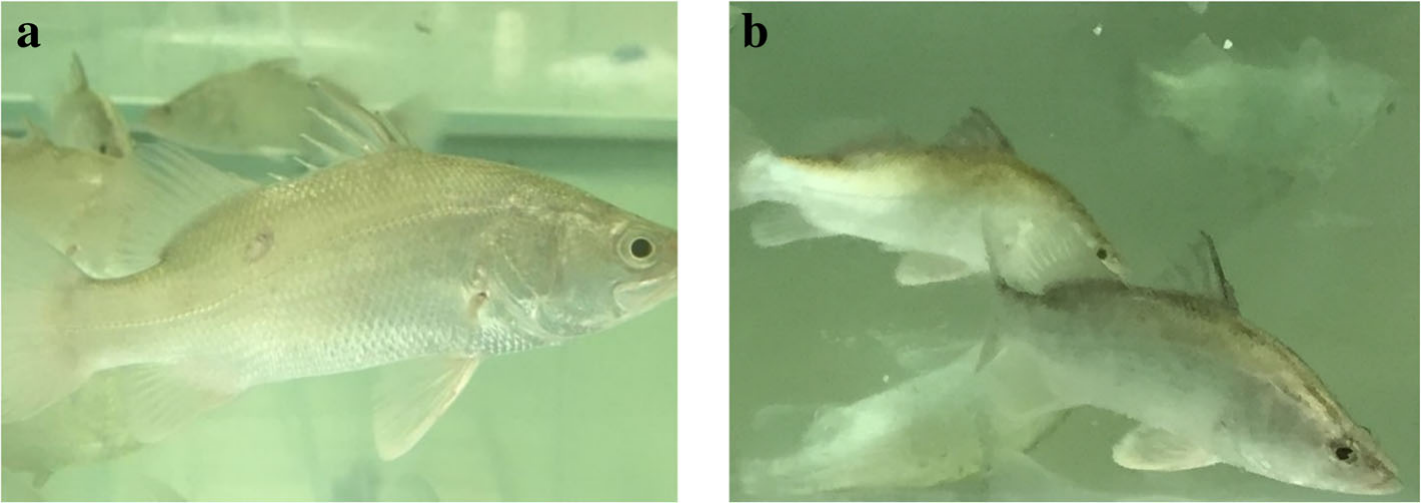
Experimental challenge of naïve fish with LCBV. **a** The fish experimentally injected with LCBV showed “white patch” lesions during the first week of infection; **b** Fish in the control group showed no lesion

An experimental fish challenge study was done to check for fulfilment with Koch’s postulates. The supernatant from case A17/2/15 infected BF-2 cell culture (6th passage) was injected via the intra-peritoneal route into Asian seabass fingerlings to observe if infected fish would display similar clinical signs of disease. All of the naïve fish that were injected with the control supernatant remained aclinical while several fish injected with infected BF-2 culture showed clinical signs of localized scale loss at 3 days’ post-injection. A few of the inoculated fish exhibited “white patch” lesions on the body during the first week of infection with lesions disappearing in the later phase of infection, thereby indicating that the fish may successfully mount a protective immune response against LCBV (Fig. 4a and b). The mortality rate was not significantly different in fish infected with LCBV and fish in the control group.

Furthermore, the organ homogenates of the LCBV-infected fish were tested positive by reverse-transcription polymerase chain reaction (RT-PCR) using primer set Seg B 1F and 1R (data not shown), while the control fish were tested negative. The organ homogenates of LCBV-infected fish were also inoculated into BF-2 cells and these cultures developed the characteristic CPE, indicative of successful virus isolation. The cell culture passages were continued for 5 passages and all the experimentally-infected samples were tested positive by RT-PCR. CPE was not evident in BF-2 cell culture when the control fish organ homogenates were used for virus isolation. This experimental fish challenge study confirmed that the novel LCBV could infect Asian seabass and be re-isolated from the infected fishes, although the natural route of virus infection remains to be studied and determined.

## Discussion

In this study, we report the first detection of LCBV isolated from diseased Asian seabass on coastal farms in Singapore in 2015. LCBV is previously undescribed and attempts to characterize the virus were performed. First, the inoculation of organ homogenates from diseased Asian seabass into BF-2 cells resulted in the appearance of CPE. Subsequent inoculation of the supernatant harvested from these infected cultures into new cell cultures maintained the similar CPE. Second, using TEM, virion-like structures were visualized in infected-cell supernatants as electron-dense particles. TEM analyses, combined with sensitivity studies to chloroform and BUDR, provided evidence that LCBV is a non-enveloped RNA virus. Third, the NGS results and phylogenetic analyses of the virus genome revealed that a novel birnavirus was isolated from the infected cell supernatants of cases A17/2/15 and A11/6/17. Furthermore, when naïve Asian seabass were injected with the supernatant from LCBV-infected BF-2 cell culture under experimental conditions, similar clinical signs such as the “white patch” lesions were reproduced. In addition, the same viral gene sequences could be amplified from LCBV-infected fish and the same virus can be re-isolated in BF-2 cell line, substantiating the fulfilment of Koch’s postulates for this virus.

Birnavirus had been known to infect fish as early as the 1940s and the virus can be transmitted both vertically via the eggs and horizontally via water [17, 18]. In addition, birnavirus was first reported to be possibly associated with mortalities in hatchery-reared seabass fry in 1983 [19]. As described previously, mortality rates of aquatic birnaviruses associated with disease outbreaks can be quite variable [17]. In our study, there have been detections of LCBV in Asian seabass, including case A11/6/17 in several coastal fish farms in Singapore since the first detection in 2015. The low mortality rate in the fish farms and experimental fish challenge study suggests that the virus may not be significantly virulent and this could help to explain the lack of significant histopathology findings to conclusively support a viral infection.

Out of the four genera of the *Birnaviridae* family, the *Aquabirnavirus* and *Blosnavirus* genera contain birnaviruses that infect aquatic species. The most well-known fish birnavirus species and the most well-characterised member in the *Aquabirnavirus* genus is infectious pancreatic necrosis virus (IPNV). This is because IPNV has a very broad host range and can infect many different species of fish as well as molluscs and crustaceans, causing necrotic lesions in the pancreas and kidney [20]. IPNV is hence classified as a World Organisation for Animal Health (OIE)-listed disease-causing virus [21]. After IPNV was discovered, several other marine birnaviruses including a birnavirus isolated from yellowtail fish have been reported [22–24].

IPNV-infected fish typically demonstrates clinical signs including darkening of the skin, abdominal swelling and abnormal behavioural signs ranging from lethargy to short bursts of erratic swimming [15]. Significant necrotic changes can also be found in the pancreas, kidney, intestinal mucosa and spleen of IPNV-infected fish [15]. On the other hand, only “white patch” lesions on the body were observed from the LCBV-infected fish which is different from a typical aquatic birnavirus infection such as IPNV. Moreover, the “white patch” lesions on the LCBV-infected fish disappeared during the later phase of infection in the experimental fish challenge study, suggesting that the fish may mount an effective immune response against the virus without significant mortality. Further studies need to be carried out to determine the natural route of virus infection and characterize the clinical signs of fish infected by LCBV.

There also exists a low sequence similarity between LCBV and the known birnaviruses within the *Aquabirnavirus* genus, making PCR detection of LCBV using published primers difficult. This may help to explain why PCR detection of LCBV using known primers was unsuccessful [25, 26]. Therefore, NGS was employed to obtain the virus genome sequence from the virus isolated from BF-2 cell culture. Based on phylogenetic analyses of the gene sequences obtained by NGS, LCBV is found to be most closely related to BSNV in the *Blosnavirus* genus and separated from IPNV and other viruses in the *Aquabirnavirus* genus. This result is in agreement with the sequence analysis whereby LCBV shared the highest level of sequence similarity with BSNV as compared to viruses in the *Aquabirnavirus* genus. As BSNV has been classified under the genus *Blosnavirus* by the International Committee on Taxonomy of Viruses, LCBV could be classified under the genus *Blosnavirus* together with BSNV [27]. LCBV could also be under a new genus of the *Birnaviridae* family, separated from *Blosnavirus*. This hypothesis requires further confirmation through characterization of LCBV and additional experiments such as 5′ and 3′ rapid amplification of cDNA ends (RACE) can be carried out to obtain the terminal sequences in the 2 genome segments for more in-depth phylogenetic analyses.

This work re-emphasizes the importance of surveillance programs to keep up with emerging pathogens. With the increased surveillance on marine fish samples, it may be possible to derive the prevalence of the viral disease in our local fish population and may reflect the virus epidemiology in the neighbouring regions. This work also calls for further studies on serotyping and the potential transmission routes of the virus. Taken together, these observations may have important implications for disease containment strategies and be exploited for potential fish vaccine development.

## Conclusion

A novel birnavirus LCBV having a non-enveloped RNA genome and an icosahedral shape of ~ 50 nm in diameter was detected and characterized using molecular and biochemical methods through disease investigations of Asian seabass from coastal fish farms in Singapore. It was found that LCBV could infect and induce CPE in BF-2 cells. Similar clinical signs can be observed in an experimental fish challenge study and the fish can recover from the mild lesions caused by LCBV infection. NGS was also used to characterize the virus genome and LCBV was shown to be more closely related to BSNV rather than IPNV in phylogeny studies. NGS, when complemented with good bioinformatics analyses, served as a useful tool for genome annotation of unknown viruses. The sequences obtained from NGS could be used for the development of detection strategy using PCR, which is important and can enable timely disease investigation to be carried out and disease containment plans can be executed quickly.

## Abbreviations

AVA: Agri-Food and Veterinary Authority of Singapore
BF-2: Bluegill Fry cell line
BSNV: Blotched Snakehead Virus or *Channa lucius* virus
BUDR: 5-bromo-2′-deoxyuridine
CCD: Charge-Coupled Device
CPE: Cytopathic effect
HEPES: 4-(2-hydroxyethyl)-1-piperazineethanesulfonic acid
i.p.: intra-peritoneally
IBDV: Infectious Bursal Disease Virus
IPNV: Infectious Pancreatic Necrosis Virus
LCBV: *Lates calcarifer* Birnavirus
LCHV: *Lates calcarifer* Herpes Virus
MEM: Minimal Essential Media
NGS: Next-Generation Sequencing
OIE: World Organisation for Animal Health
PBS: Phosphate Buffered Solution
PCR: Polymerase Chain Reaction
RACE: 5′ and 3′ rapid amplification of cDNA ends
RT-PCR: Reverse-Transcription Polymerase Chain Reaction
SDDV: Scale Drop Disease Virus
TCID50: 50% Tissue Culture Infective Dose
TEM: Transmission Electron Microscope
TNE: 10 mM Tris-HCL, 100 mM NaCL, 10 mM EDTA, pH 7.5
